# Recurrent massive bleeding from a small intestinal arteriovenous malformation after surgery for biliary atresia in an infant: a case report and literature review

**DOI:** 10.1186/s40792-021-01288-3

**Published:** 2021-09-08

**Authors:** Wataru Kudo, Katsunori Kouchi, Ayako Takenouchi, Aki Matsuoka, Kiyoaki Yabe, Tadao Nakazawa, Atsuko Hasegawa

**Affiliations:** 1 Department of Pediatric Surgery, Tokyo Women’s Medical University Yachiyo Medical Center, 477-96 Ohwada-shinden, Yachiyo, Chiba 276-8524 Japan; 2 Department of Pathology, Tokyo Women’s Medical University Yachiyo Medical Center, 477-96 Ohwada-shinden, Yachiyo, Chiba 276-8524, Japan

**Keywords:** Small intestinal arteriovenous malformation, Small intestinal bleeding, Intraoperative endoscopy, Frozen section analysis, Infant, Biliary atresia

## Abstract

**Background:**

Small intestinal arteriovenous malformation (AVM) can cause bleeding. Most small intestinal AVMs occur during adulthood, rarely in infancy. We report a case of an infant with hemorrhage due to small intestinal AVM early and recurrently after Kasai portoenterostomy (PE) for biliary atresia (BA).

**Case presentation:**

A 51-day-old male infant was admitted to our institution for obstructive jaundice. Laparotomic cholangiography revealed BA (IIIb1μ), and Kasai PE was performed at 60 days of age. On postoperative day 17, he developed massive melena and severe anemia. Contrast-enhanced computed tomography (CT) revealed that the jejunum around the PE site was strongly enhanced with enhancing nodules in the arterial phase, and a wide area of the Roux limb wall was slightly enhanced in the venous phase. As melena continued, emergency laparotomy was performed. There were no abnormal macroscopic findings at the PE site except for a clot in the Roux limb 5 cm away from the PE site, and the Roux limb was resected 5 cm. On further investigation, a red spot was detected on the jejunal serosa 30 cm away from the Roux-en-Y anastomosis site. PE and wedge resection for the red spot were performed. Histopathologically, both specimens indicated AVM. He was jaundice-free 65 days after the first surgery. However, at 7 months of age, he developed massive melena again. Contrast-enhanced CT and upper gastrointestinal endoscopy revealed no bleeding lesions. Hemorrhagic scintigraphy showed a slight accumulation at the hepatic hilum prompting an emergency surgery. Intraoperative endoscopy detected a bleeding lesion at the PE site, and the Roux limb was resected (approximately 6 cm). Intraoperative frozen section analysis of the stump of the resected jejunum revealed no abnormal vessels. PE was performed, and permanent section analysis revealed an AVM in the resected jejunum. The postoperative course was uneventful without re-bleeding.

**Conclusions:**

We experienced a case of recurrent massive bleeding from small intestinal AVM in an infant after surgery for BA. Intraoperative endoscopy and frozen section analysis helped identify the bleeding lesion and perform a complete resection of the small intestinal AVM, even after surgery, in the infant.

## Background

After Kasai portoenterostomy (PE) for biliary atresia (BA), most of the causes of gastrointestinal (GI) bleeding are esophageal varices, which form and bleed as liver cirrhosis and portal hypertension progress. In a previous report [1], GI bleeding due to esophageal varices occurred in 22% of the patients at a median age of 17 months (4 months to 5 years). Meanwhile, early postoperative GI bleeding as a surgical complication is rare and occurs in 3.1–5.4% of the patients [2, 3].

Herein, we report a rare case of an infant who experienced bleeding from the Roux limb very early after Kasai PE for BA, before progression of portal hypertension; the cause was identified as an intestinal arteriovenous malformation (AVM).

## Case presentation

A 51-day-old boy was referred to our institution with jaundice and acholic stool. Physical examination results were unremarkable, except for skin jaundice. Laboratory analysis revealed total bilirubin, direct bilirubin, and γ-GTP levels of 6.7 mg/dL (normal: 0.1–1 g/dL), 5.2 mg/dL (normal: 0.1–0.4 g/dL), and 1,295 IU/L (normal: 11–58 IU/L), respectively. These results were indicative of obstructive jaundice and liver disorder, and the patient was admitted. At 60 days of age, laparotomic cholangiography revealed BA (IIIb1μ), and Kasai PE was performed.

On postoperative day 17, the patient suddenly developed massive melena and was determined to have severe anemia (hemoglobin level: 6.5 g/dL). His coagulation data were normal with a prothrombin time-international normalized ratio of 1.26 (normal: 0.80–1.20) and activated partial thromboplastin time of 37.1 s (control time: 28.0 s).

**Fig. 1:**
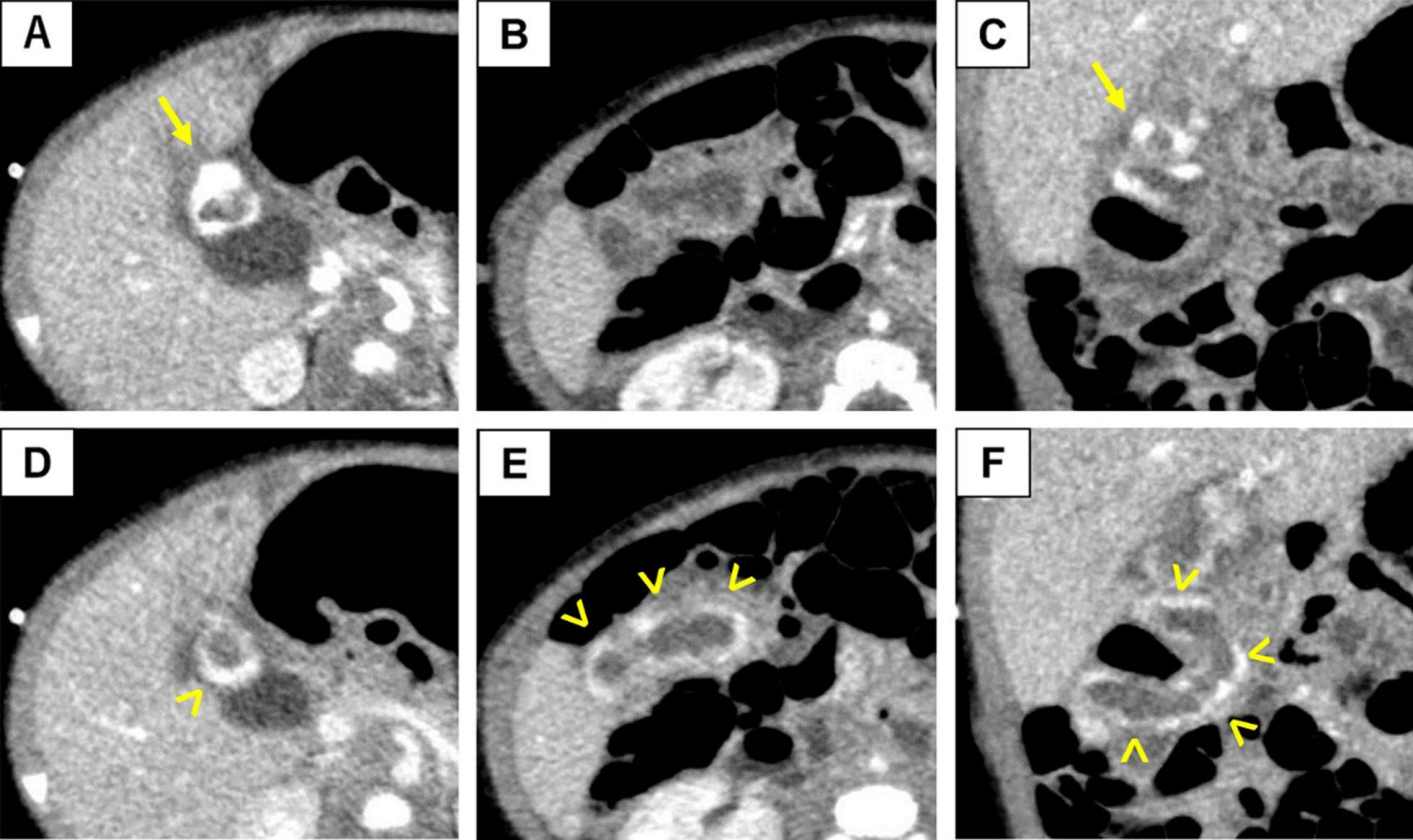
Abdominal contrast-enhanced computed tomography performed at 77 days of age. In the arterial phase (**A** and **B** on axial, **C** on sagittal), nodule-like lesions were strongly enhanced (arrow) near the portoenterostomy site. In the venous phase (**D** and **E** on axial, **F** on sagittal), enhanced lesions spread to the Roux limb (arrowhead). No dilated vessels and extravasation into the intestinal tract were observed

Contrast-enhanced computed tomography (CT) revealed that the jejunum near the PE site was partially and strongly enhanced with enhancing nodules in the arterial phase, and a wider area of the imported jejunal wall was enhanced in the venous phase (Fig. 1). Extravasation into the intestines was not detected.

Because the hemorrhage did not stop, an emergency laparotomy was performed for hemostasis. No bloody ascites were observed. The PE site was apparently normal, and there were no varices at the surface of the intestine. The anastomosis of PE was detached, and no obvious bleeding or lesions were observed at the site of the anastomotic jejunum. A 5-cm incision was made vertically from the anastomotic jejunal site, and a blood clot was found. The Roux limb was resected 5 cm from the anastomotic site. Further investigation of the small intestines revealed a red spot (suspected to be an AVM) on the jejunal wall, which was located 30 cm from the Roux-en-Y anastomosis. The red spot lesion was wedge resected, and PE was performed.

**Fig. 2:**
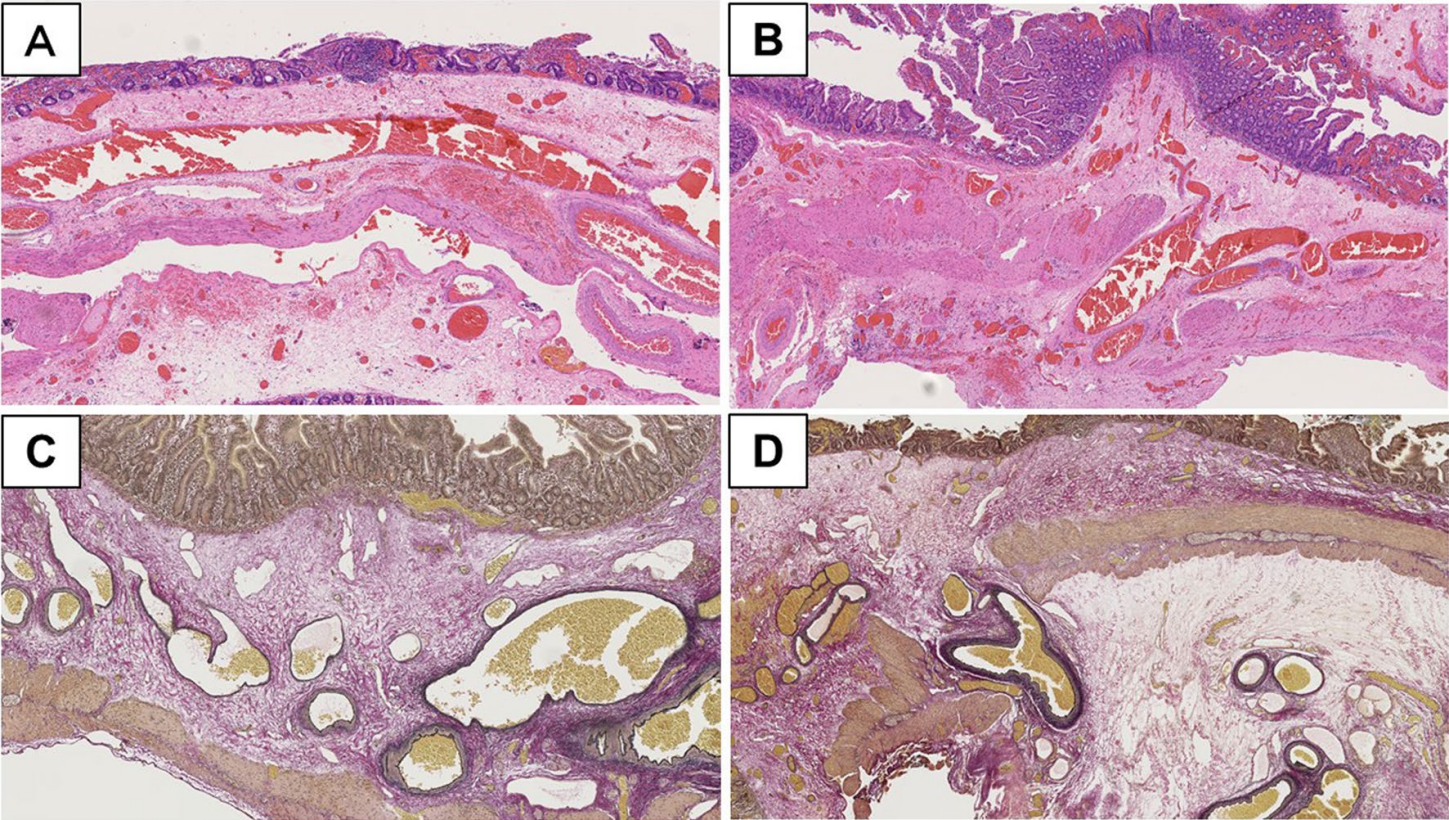
Representative microphotographs of jejunal AVM at the firstly resected specimen. **A** Remarkably dilated blood vessels aggregated in submucosal tissue. **B** An abnormal vessel penetrated and separated muscularis propria. **C**, **D** Elastica van Gieson staining disclosed that elastic fibers with variable thickness were included in these vessels of AVM. **A**, **B** hematoxylin and eosin; **C**, **D** Elastica van Gieson. Magnifications: **A**  ×100, **B ** ×100, **C**  ×200, **D**  ×100. AVM: arteriovenous malformation

Macroscopic examination of the excised Roux limb specimen showed no abnormal findings, such as ulcer and tumor. In contrast, microscopic examination revealed that the AVM comprised aggregates of abnormally shaped vessels showing hemorrhage (Fig. 2). The lesions were distributed in the submucosal tissue, muscularis propria, and subserosal tissue. These abnormal vessels markedly dilated and occasionally branched with hemorrhage in lamina propria (Fig. 2A and B). Each vessel wall showed viable thickness and contained abundant elastic fibers in the Elastica van Gieson-stained sections (Fig. 2C and D). The abnormal vessels displayed various morphologies that mimicked veins or arteries. In addition, some vessels were difficult to interpret due to vein-like or artery-like appearance. Some AVMs penetrated and fractured the muscularis propria (Fig. 2D).

The patient was jaundice-free at 125 days of age and was discharged at 145 days of age.

**Fig. 3:**
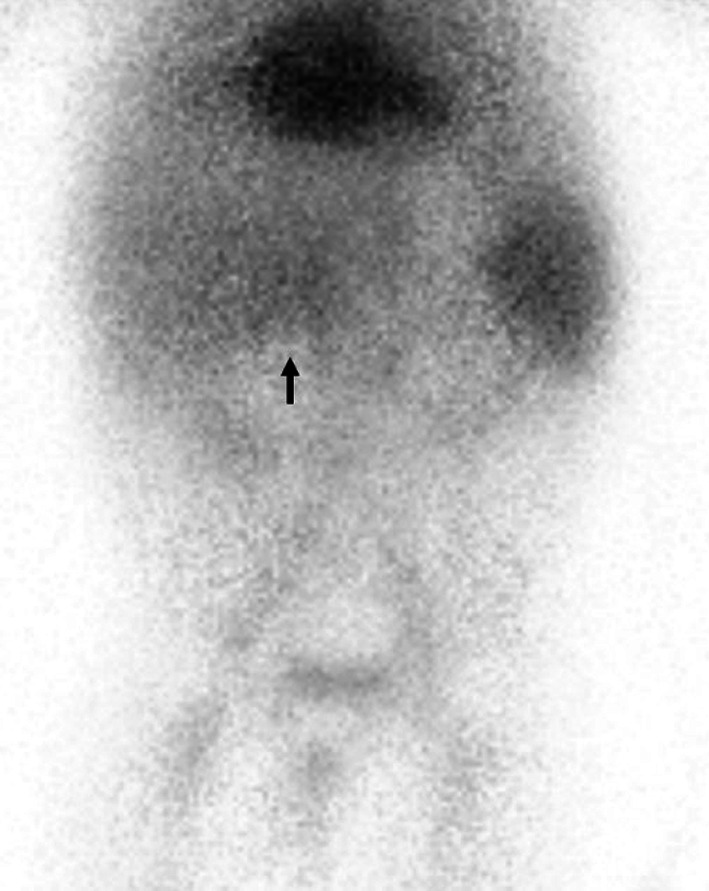
Hemorrhage scintigraphy. Technetium-99 m-human serum albumin-diethylenetriaminepenta-acetic acid (740 MBq) was administered intravenously. The concentration at the portoenterostomy site (arrow) peaked 24 min after injection. After 60 min, the concentration did not migrate

**Fig. 4:**
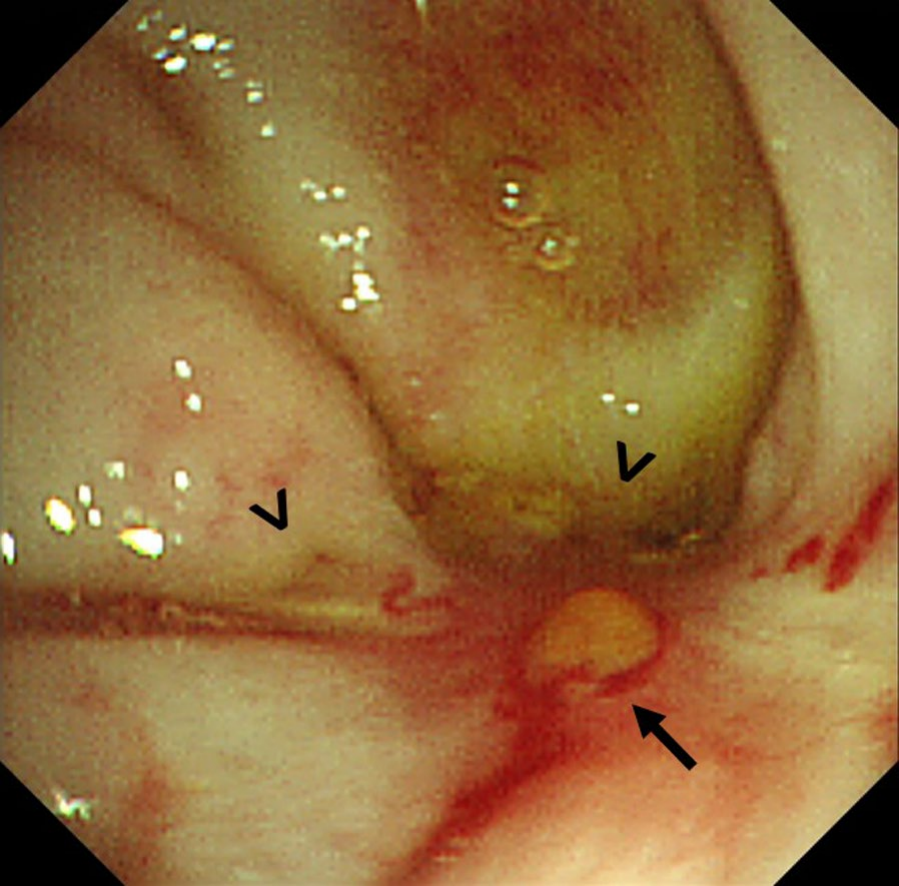
Intraoperative endoscopic examination. The endoscope reached the portoenterostomy site and revealed the left and right bile outflow orifices (arrowheads) and the bleeding lesion (arrows)

**Fig. 5:**
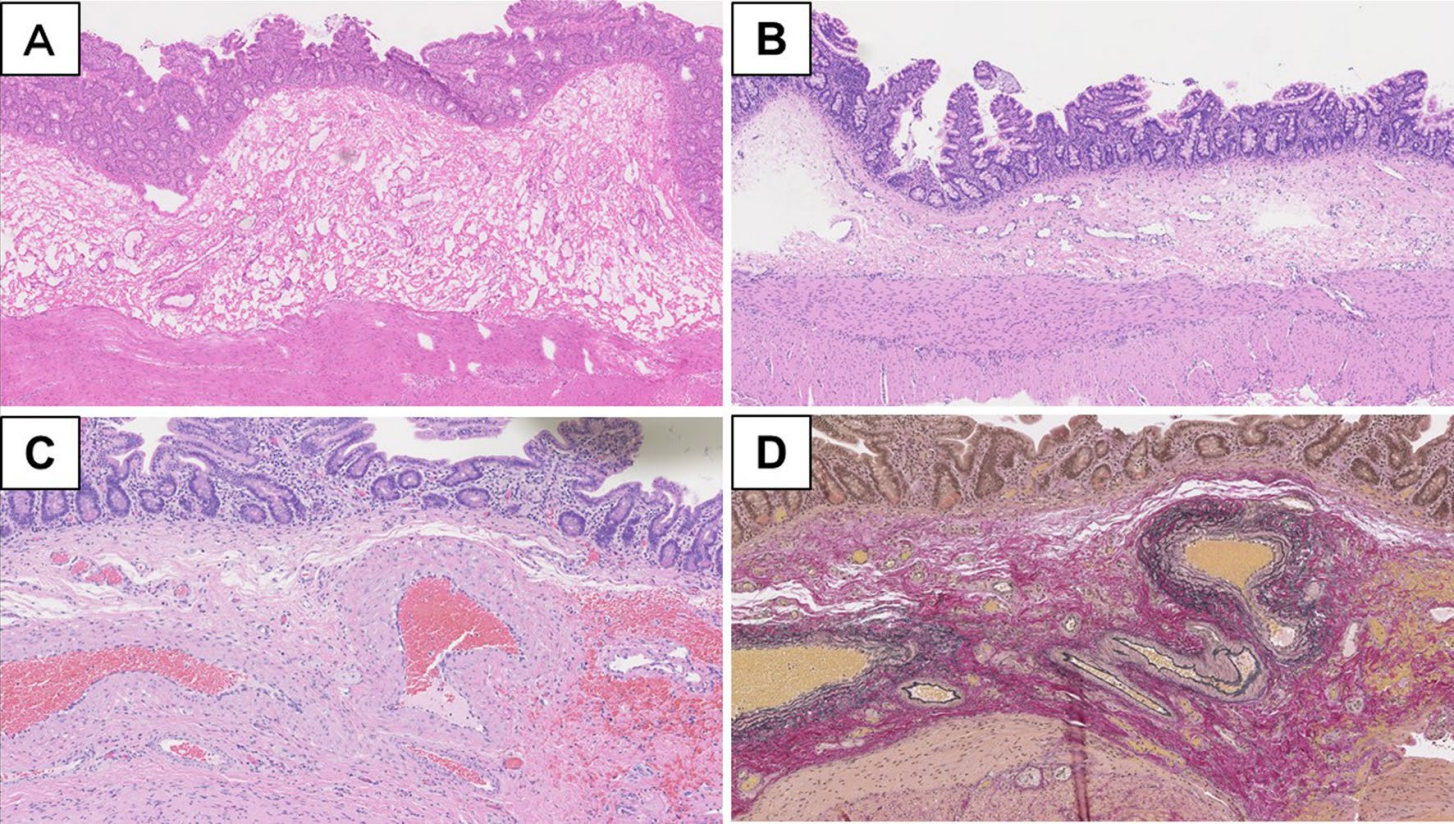
Representative microphotographs of residual AVM in jejunum at the secondarily resected specimen. No AVM in the surgical margin was confirmed at frozen (**A**) and permanent (**B**) sections. **C**, **D** A similar AVM was observed in the submucosal tissue in the jejunum around the bleeding point at the PE site. **A**–**C** hematoxylin and eosin; **D** Elastica van Gieson. Magnifications: **A**  ×100, **B**  ×100, **C**  ×200, **D**  ×200. AVM: arteriovenous malformation

At 7 months of age, massive melena was observed, and he was re-admitted. Contrast-enhanced CT revealed no abnormal findings, and upper GI endoscopy revealed no esophageal or gastric varices. Hemorrhagic scintigraphy detected a slight accumulation at the hepatic hilum; thus, hemorrhage from the PE site was suspected (Fig. 3). As a residual lesion of the AVM was a possible source of the bleeding, we consulted a pathologist preoperatively, planned a frozen section analysis (FSA) for intraoperative margin assessment, and performed an emergency surgery. The jejunal Y-shaped anastomosis was detached, and the endoscope was inserted. Endoscopy detected bleeding lesions with an erosion of the Roux limb at the PE site (Fig. 4); there were no other findings in the small intestine. The PE anastomosis and its mesentery had no apparent abnormalities, such as varices. The anastomosis was detached, and the Roux limb was resected approximately 6 cm from the anastomosis. FSA for the stump of the anal side of the resected jejunum, performed by a pathologist, revealed no vessel abnormality in the submucosa (Fig. 5A). PE and Y-shaped anastomoses were performed.

Regarding the specimen collected during the second emergency operation, no obvious AVM was microscopically detected in the surgical stump of the Roux limb, even in the permanent sections (Fig. 5B). Remnant AVMs were seen with serosal fibrosis and suture granuloma (Fig. 5C and D). The AVM was localized in submucosal tissue with hemorrhage in lamina propria (Fig. 5C). The presence of elastic fibers was confirmed in these vessel walls in the Elastica van Gieson-stained section (Fig. 5D).

The postoperative course was uneventful with no re-bleeding, and the patient was discharged on postoperative day 15.

## Discussion

Small intestinal bleeding accounts for approximately 5% of all GI bleeding cases and is rarely caused by an AVM [4]. Most small intestinal AVMs occur during adulthood and are very rare in early childhood [4, 5]. Etiologically, AVMs are mostly congenital [6]. In some cases, diagnosis and treatment are difficult; however, emergency procedures are required if a massive hemorrhage develops.

To consider small intestinal AVM, we planned to review the literature. The number of case reports of small intestinal AVM published in English, which had detailed information, was small [4]. We decided that it would be better to evaluate many cases to comprehensively understand the etiology, pathogenesis, and treatment of small intestinal AVMs. For this reason, we conducted a literature survey of Japanese case reports and reviews on small intestinal AVMs. In this search, we used the words "arteriovenous malformation" and "intestine" in Japanese for "Ichushi-Web" and in English for "PubMed". Following the literature search, we found 198 cases of small intestinal AVMs reported during 1976–2020, including our present case. A summary of the clinical characteristics of these cases is presented in Table 1. The median age was 59 years; the current case, at 2 months old, is the youngest. Only eight cases (4.0%) were under 18 years of age as pediatric small intestinal AVMs are rare. Most lesions were isolated (173 cases, 87.4%), and jejunal lesions were more common than ileal lesions (98 cases vs. 62 cases). Multiple or extensive lesions were observed in 25 cases (12.6%). Sixty-six cases had complications (33.3%); liver disease was the most common complication observed [*n* = 31, 15.7%; cirrhosis (*n* = 15) and acute or chronic hepatitis (*n* = 8)]. Besides our case, only one other case underwent Kasai PE for BA and developed bleeding at 1 year and 2 months of age; two lesions unrelated to the Roux-en-Y structure were observed in the small intestine [7]. It is assumed that increased portal pressure due to liver disease increased the risk of bleeding from the small intestinal AVM.

**Table 1:** Clinical characteristics of the 198 Japanese cases of small intestinal arteriovenous malformations

Characteristic	Value
Age -median (range)	59 yr (2 mo–95 yr)
Sex -no. (%)	
Male	115 (58.1)
Female	83 (41.9)
Lesion number and location -no. (%)	
Isolated lesion	173 (87.4)
Jejunum	98 (49.5)
Ileum	62 (31.3)
Small intestine	13 (6.6)
Multiple or diffuse lesion	25 (12.6)
Diffuse lesion	3 (1.5)
Multiple lesions within the small intestine	7 (3.5)
Coexisting lesion other than small intestine^a^	13 (6.6)
Large intestine	10 (5.1)
Duodenum	2 (1.0)
Stomach	1 (0.5)
Pancreas	2 (1.0)
Liver	1 (0.5)
Coexisting conditions^a^ -no. (%)	
Any	66 (33.3)
Liver disease	31 (15.7)
Cardiovascular disease	20 (10.1)
Diabetes mellitus	12 (6.1)
Renal disease	9 (4.5)
Other	21 (10.6)
Examination, *n* (positive^b^/total^c^)	
Angiography	(124/133)
Contrast-enhanced CT	(40/66)
Endoscopy^d^	(59/60)
Treatment, *n* (%)	
Operation	139 (70.2%)
Operation followed IVR^e^	28 (14.1%)
IVR^e^	19 (9.6%)
Re-bleeding	11
Perforation	3
Endoscopic hemostasis	5 (2.5%)
Re-bleeding	9
Conservation	7 (3.5%)

^a^Some patients had several diseases or lesions

^b^Total number of performed examinations

^c^Number of examinations with positive findings

^d^Number of examinations including capsule endoscopy, small bowel endoscopy, and intraoperative endoscopy

^e^Interventional radiology

Various imaging devices have been used to localize the lesions. Traditionally, selective mesenteric angiography has been the most reliable method for diagnosing small intestinal AVMs and can mark lesion sites during the examination [8]. Recently, multi-detector CT has been used to identify these lesions [9]. Our literature review of the 198 cases in Japan revealed that contrast-enhanced CT was performed in 66 cases: CT findings were observed in 40 cases (Table 1). The characteristic findings on the CT images were: (a) hypervascularity nodules and thickening of the small intestinal wall; (b) strong enhancement in the arterial phase and an early return in the venous phase; (c) hyperplastic or dilatation of the arteries and veins; and (d) extravasation into the intestinal tract. These findings are similar to the angiographic findings [10]. Furthermore, diagnosis using capsule or double-balloon endoscopy has been reported since 2000 [11–15]. In almost all cases that underwent endoscopy, including capsule endoscopy, small bowel endoscopy, and intraoperative endoscopy, the lesion could be identified (Table 1).

Recently, the effectiveness of a combination of indocyanine green (which can visualize the intestinal blood flow) and angiography has been reported [4, 16]. Regarding treatments in the Japanese literature review, while endoscopic or endovascular treatments are available, 3 of the 5 reports on endoscopic treatment alone and 12 of the 19 reports on endovascular treatment alone had recurrence or perforation and required surgery. Therefore, these treatments were uncertain for hemostasis; surgical resection is the most reliable option.

Our patient is the youngest reported case of a small intestinal AVM characterized after PE for BA. We inferred the mechanism of early bleeding in our patient as follows: (1) transection of the jejunum for Roux-en-Y reconstruction blocked the blood circulation in the intestinal wall; (2) the AVM was coincidentally present near the transected site, and (3) the vessel pressure of the AVM was elevated, which caused bleeding. Additionally, although we did not measure portal pressure to prove portal hypertension at this time, BA resulted in increasing venous return pressure in the portal system, which may still have been involved in the bleeding. The bleeding lesion was resected during surgery after the first bleeding; however, the AVM lesion probably remained near the resected margin and caused the bleeding to recur.

The most common causes of massive bleeding after BA surgery are varicose veins or mucosal congestion due to portal hypertension. However, in the absence of endoscopic findings indicative of varicose veins or mucosal congestion, and contrast-enhanced CT shows characteristic findings of AVM, intestinal AVM should be considered the differential diagnosis. Whether or not the identified site is prone to varicose veins may also help in the diagnosis. The most challenging issue in our case was that neither abnormalities were found on serosa nor mucosa on AVM lesion. During Kasai PE and the first emergency operation, the serosa of the small intestine was normal in appearance except for a red spot. At the second bleeding, we detected the bleeding point by intraoperative endoscopy; however, the mucosa near the bleeding point appeared normal, and no other abnormalities were found in the small intestine on endoscopy or macroscopic investigation. The reported devices, such as double-balloon endoscopy and capsule endoscopy, could not detect the extent of AVM lesions. Intraoperative FSA has been used to determine the extent of resection for various diseases in general [17].

Histopathological findings of small intestinal AVM are characterized by aberrant vessels with varying thickness, and the connections between arteries and veins lack a capillary network; these abnormal vessels are mainly in the submucosa and spread to the subserosa [6, 18]. Because these histopathological findings of the intestinal AVM are not highly complicated, intraoperative FSA was valuable and helpful in determining the extent of intestinal AVM resection.

## Conclusions

We experienced an infant case who developed recurrent massive bleeding from small intestinal AVM after Kasai PE for BA. Small intestinal lesions, especially in Roux limbs, are difficult to reach endoscopically. Moreover, it is difficult to evaluate the spread of AVMs. Intraoperative endoscopy and FSA are useful for the complete resection of small intestinal AVMs, even in infants or after surgery.

## Data Availability

The datasets used and/or analyzed during the current study are available from the corresponding author on reasonable request.

## Abbreviations

AVM: Arteriovenous malformation
BA: Biliary atresia
PE: Portoenterostomy
FSA: Frozen section analysis
GI: Gastrointestinal
CT: Computed tomography
